# Expression of suppressor of cytokine signaling 3 (SOCS3) and interleukin-6 (-174-G/C) polymorphism in atopic conditions

**DOI:** 10.1371/journal.pone.0219084

**Published:** 2019-06-28

**Authors:** Arooma Jannat, Maryam Khan, Maria Shabbir, Yasmin Badshah

**Affiliations:** Atta-ur-Rahman School of Applied Biosciences (ASAB), National University of Sciences and Technology (NUST), Islamabad, Pakistan; Université Paris Descartes, FRANCE

## Abstract

Hypersensitivity of the immune system is caused by elevated immunoglobulin E (IgE) levels in the serum, in response to a discrete allergen leading to allergic reactions. IgE-mediated inflammation is regulated by the cascade of defense related signaling molecules including interleukin-6 (IL-6) that plays pivotal role in the survival and maturation of mast cells during an allergic reaction. IL-6 mediated defense responses are tightly regulated by Suppressor of Cytokine Signaling 3 (SOCS3), an inhibitory molecules of Janus Kinase-Signal Transducers and Activators of Transcription (JAK-STAT) signaling, in a negative feedback mechanism. The given study focuses on the assessment of crosstalk between SOCS3 and IL-6 to unravel the molecular significance of SOCS3 and IL-6 in the diagnosis and prognosis of allergy. The expression study of SOCS3 through real-time PCR analysis revealed, a 5.9 mean fold increase in SOCS3 expression in atopic cases in comparison to control cases. Moreover, IL-6 has, also, been found significantly enhanced in the serum level of atopic cases (26.4 pg/ml) as compared to control cases (3.686 pg/ml). Female population was found to be at a higher risk to develop atopic condition than male population as females exhibited higher expression of both SOCS3 and IL-6 than males. Furthermore, the polymorphic study of IL-6 promoter region (IL-6 174-G/C) in atopic population has reasserted the importance of SOCS3 and IL-6 in the diagnosis and prognosis of allergy. Expression of SOCS3 and IL-6 serum levels were found to be highly correlated. Therefore establishing the role of IL-6 (-174-G/C) polymorphism on the expression of SOCS3 and IL-6 in atopic cases. Notably, the study established SOCS3 and IL-6 as potential targets for the diagnosis/prognosis of allergy and for the development of reliable therapeutic strategies to control atopic conditions in the near future.

## Introduction

Hypersensitivity of the immune system, due to elevated level of immunoglobulin E (IgE), instigates the allergic inflammation that leads to atopic conditions, including rhinitis, conjunctivitis, asthma, food allergy and anaphylaxis, after the exposure to a specific associated allergen [1]. The incidence of allergy is increasing day by day in the various regions of the world. According to American Academy of Allergy, Asthma, and Immunology, 10–40% of the world population has been reported with allergen sensitization to foreign antigens [2]. IgE-mediated inflammation, triggered by IgE-specific antigen, is regulated by the cascade of defense signaling involving FcεR (high affinity receptor of IgE) on the surface of mast cells [3]. Cross-talk through the activity of pro-inflammatory cytokines such as interferon γ (IFN- γ), interleukin-6 (IL-6), IL-13, IL-5, IL-4, granulocyte macrophage colony stimulating factor (GM-CSF) and other chemokines is essential to regulate allergic responses [2, 4–10]. This vast spectrum of pro-inflammatory cytokines implies that it is a Th2 cell mediated response that leads to late phase allergic response [11–14].

IL-6 is a crucial immune system regulator that is involved in the survival and maturation of mast cells; thereby it is associated with the prognosis of allergy [15–18]. IL-6 is a potent inducer of Janus Kinase-Signal Transducers and Activators of Transcription (JAK-STAT) signaling cascade. IL-6 initiates JAK-STAT signaling cascade and the expression of Suppressor of Cytokine Signaling 3 (SOCS3), a signaling molecule which regulates the immune responses to inflammation and infection [19]. SOCS3 controls the IL-6 mediated signaling cascade through the negative feedback mechanism [20]. The increased expression of SOCS3 has been observed in the patients with allergic conditions. Moreover, the previous studies suggested that silencing or deletion of SOCS3 in the animal model leads to the aggravation of airway hyper-responsiveness and other inflammatory conditions. Thereby, it is implying that SOCS3 has a protective role in allergic conditions by mediating the over-expression of IL-6 and STAT3 and suggesting that allergic inflammation is strictly regulated by IL-6/STAT3/SOCS3 axis [21–26]. SOCS3 suppresses IL-6 activity, however, higher and persistent exposure of IL-6 obstructs the activity of SOCS3 [21].

The 174-promoter region of IL-6 gene harbors a functional polymorphism, G C (rs1800795), which alters the IL-6 serum levels [27]. The genotype GG and GC have been attributed as high IL-6 producing genotypes whereas CC has been regarded as a low producer of IL-6. Previous studies revealed that GG and GC are prevalent in cases with allergy in comparison to CC genotype [28–31]. The association of IL-6 (-174-G/C) polymorphism has been established with various inflammatory conditions and has been associated with the prognosis and pathogenesis of the inflammatory disorders [32, 33]. SOCS3 activity is strictly controlled by IL-6 thus any alteration in IL-6 serum levels may affect SOCS3 expression and activity [34]. SOCS3 is known to mediate allergic response through Th2 activity while its attenuation aggravates the allergic conditions thus, establishing its protective role against allergic reaction [21, 25, 26, 35]. The study was designed to evaluate the impact of IL-6 (-174-G/C) polymorphism and IL-6 on SOCS3 expression in various atopic conditions in order to assess its potential as a diagnostic/prognostic marker. Allergy is a gender biased condition and is known to affect females more than male so we further analyzed the levels of SOCS3 in male and female atopic cases to establish a molecular basis for the claim.

## Materials and methods

### Sample collection

Prevalence case-control study model based on single population was adopted for the given study after the approval from Institutional Review Board of Atta-ur-Rahman School of Applied Biosciences (IRB-ASAB). All the procedures were performed by the guidelines provided by ethical review board. Sample size determination and power analysis were conducted by adopting method already described by Charan & Biswas, 2013 [36]. A total of 159 atopic cases and 152 control cases were recruited from population of twin cities of Pakistan (Islamabad and Rawalpindi) during the time-frame of February-March 2016 on the basis of exclusion and inclusion criteria. Patients with confirmed allergic status were included in the study; cases with negative skin prick test were excluded from the study. Patients were stratified on the type of allergy. The blood and serum samples were collected after an abridged clinical history form and a written consent.

### Genotyping

DNA was isolated using standardized sodium dodecyl sulfate (SDS) mediated cell lysis, *Protinase K* digestion, phenol-chloroform extraction and ethanol precipitation from the whole blood [37]. Genotyping of IL-6 (-174-G/C) polymorphism was done through Amplification-Refractory Mutation System PCR (ARMS-PCR) using the primer sets (Macrogen, Seoul, South Korea) already reported in Badshah et. al., 2018 [32]. The results were analyzed using 2% agarose gel.

### Quantification of SOCS3 and IL-6

The expression of SOCS3 was quantified by real-time PCR technique. The cDNA was synthesized from mRNA extracted from the peripheral white blood cells using TRIzol reagent manufactured by Thermo Fisher Scientific (Waltham, MA, USA) through the method reported by Simms et, al., 1993 with modifications [38]. The sequences of the primer sets (Macrogen) were retrieved from Komyod et. al., 2007 for PCR analysis [39]. The relative expression of SOCS3 was calculated using Livak method (2^-ΔΔCT^) while beta-actin gene was used as the housekeeping gene for the normalization of SOCS3 expression [40]. Human IL-6 ELISA Kit (ab46027) (Abcam, Cambridge, UK) was used to determine the levels of IL-6 in the serum of both the control and patient group according to the manual instructions.

### Statistical analysis

The experimental data was organized using Microsoft office 2013 Excel (Rehmond, WA, USA) while GraphPad Prism version 5.01 (GraphPad Software Inc., San Diego, CA, USA) was used for both, descriptive and inferential statistical analysis. Chi square and *Fisher’s exact* test were used to analyze IL-6 (-174G/C) polymorphism genotype distribution and the strength of association (odd ratio i.e. OR and relative risk i.e. RR) between these genotypes and allergy. Unpaired student’s *t* test was used to determine statistically significant association and correlation among the means ± standard deviation (SD) of different groups. A *p*-value of less than 0.05 was considered to be statistically significant. Pearson r value was taken as a measure for correlation.

## Results

### Demographics of the study population

Demographics of atopic and control cases has been summarized in Table 1. The mean age (±SD) group of the patients and controls includes 36.7±12.1 years and 32.6±9.3 years, respectively. The mean age group and disease prevalence did not show significant difference among control and atopic groups. However, the incidence of allergy was prevalent in the female population.

**Table 1:** Demographics of the cases under study.

Characteristics	Study Groups	
	Control (N = 152)	Patients (N = 159)
Ethnicity		
Age in years	32.6±9.3	36.7±12.1
Gender		
*Male*	68	60
*Female*	84	99
Medication	-	91
Vaccinated	-	20
Atopic Conditions		
*Food Allergies*	-	18
*Asthma*	-	65
*Rhinitis*	-	34
*Dermatitis*	-	24
*Conjunctivitis*	-	18

### Association of IL-6 (-174-G/C) polymorphism with allergy

IL-6 (-174-G/C) polymorphic study analysis depicted C allele as a risk of developing allergic conditions under study; asthma, rhinitis, conjunctivitis and food allergy except the dermatitis group which showed that G allele is risk allele for it. The frequency distribution depicted strong association of IL-6 174-G/C polymorphism with allergy and the sub-groups except dermatitis group that represented a weak association with the given polymorphism.

The homozygous genotypes, GG and CC, were prevalent in comparison to the heterozygous genotype GC in both atopic and control cases. GG genotype was prevalent in control population (59.2%) in comparison to the allergic population (40.2%) whereas CC genotype is widespread in the allergic population (35.8%) in comparison to control population (26.3%) (Table 2). The GC genotype, however, was predominant the allergic population (23.9%). Asthma patients depicted a trend similar to that of total atopic cases whereas GG was predominant in the group with rhinitis (45.8%) and dermatitis (64.2%). GC in dermatitis was equivalent to that of control population i.e. 14%. CC genotype was prevalent in the cases with food allergies (50%). GC and CC were prevalent in the conjunctivitis cases (37.5%). Allelic frequency did not vary in allergic cases; however G allele was more prevalent in control cases (66.4%). The atopic and asthma cases did not show significant difference in allelic distribution. G allele showed predominance in rhinitis (58.3%) and dermatitis (71.4%) cases whereas C allele proved to be pervasive among cases with conjunctivitis and food allergy.

**Table 2:** Genotype and haplotype frequency distribution of IL-6 174-G/C Polymorphism.

Study Group	Genotype			Allele	
	GG	GC	CC	G	C
	Number	Number	Number	Number	Number
	Percentage (%)	Percentage (%)	Percentage (%)	Percentage (%)	Percentage (%)
Control	90	22	40	202	102
(N = 152)	(59.2%)	(14.4%)	(26.3%)	(66.4%)	(33.5%)
Total Atopic Patients	64	38	57	166	152
(N = 159)	(40.2%)	(23.9%)	(35.8%)	(52.2%)	(47.7%)
Asthma	24	15	26	64	66
(N = 65)	(37.2%)	(23.5%)	(39.2%)	(49.01%)	(50.98%)
Rhinitis	15	9	10	40	28
(N = 34)	(45.8%)	(25%)	(29.1%)	(58.3%)	(41.6%)
Dermatitis	15	4	5	34	14
(N = 24)	(64.2%)	(14%)	(21%)	(71.4%)	(28.57%)
Conjunctivitis	4	7	7	16	20
(N = 18)	(25%)	(37.5%)	(37.5%)	(43.75%)	(56.25%)
Food Allergy	2	7	9	11	25
(N = 18)	(12.5%)	(37.5%)	(50%)	(31.25%)	(68.75%)

Association between IL-6 (-174-G/C) polymorphism and occurrence of allergy, for that atopic cases were stratified into sub-groups including asthma, rhinitis, dermatitis, conjunctivitis and food allergy Table 3. GG genotype has protective effect against atopic condition (Odd ratio/OR = 0.47) and sub-groups; asthma (OR = 0.4), rhinitis (OR = 0.54), conjunctivitis (OR = 0.19) and food allergy (OR = 0.08) whereas it had risk effect for dermatitis cases (OR = 1.4). CC has a protective role in dermatitis cases (OR = 0.73) but it acted as a risk genotype for atopic cases (OR = 1.5) and the sub-groups; asthma (OR = 1.86), rhinitis (OR = 1.16), conjunctivitis (OR = 1.78) and food allergy (OR = 2.8) cases. GC genotype, however, appeared to be risk genotype for atopic cases (OR = 1.85) and the sub-groups; asthma (OR = 1.77), rhinitis (OR = 2.12), dermatitis (OR = 1.18), conjunctivitis (OR = 3.76) and food allergy (OR = 3.76). The C allele acted as a risk allele for atopic cases (OR = 1.81) and the sub-groups; asthma (OR = 2.04), rhinitis (OR = 1.38), conjunctivitis (OR = 1.58) and food allergy (OR = 4.5) except dermatitis for which it had a protective effect (OR = 0.81). Whereas G allele had protective effect for atopic cases (OR = 0.55) and the sub-groups; asthma (OR = 0.48), rhinitis (OR = 0.72), conjunctivitis (OR = 0.63) and food allergy (OR = 0.22) excluding dermatitis cases (OR = 1.22) for which it acted as risk allele.

**Table 3:** Association of IL-6 174-G/C polymorphism and allergy.

SNP	Control vs Total			Control vs Asthma			Control vs Rhinitis			Control vs Dermatitis			Control vs Conjunctivitis			Control vs Food Allergy		
	^*a*^*OR*	^*b*^*RR*	*χ2*,df	^*a*^*OR*	^*b*^*RR*	*χ2*,df	^*a*^*OR*	^*b*^*RR*	*χ2*,df	^*a*^*OR*	^*b*^*RR*	*χ2*,df	^*a*^*OR*	^*b*^*RR*	*χ2*,df	^*a*^*OR*	^*b*^*RR*	*χ2*,df
	*(95%CI)*	*(95%CI)*	p-value*	*(95%CI)*	*(95%CI)*	p-value*	*(95%CI)*	*(95%CI)*	p-value*	*(95%CI)*	*(95%CI)*	p-value*	*(95%CI)*	*(95%CI)*	p-value*	*(95%CI)*	*(95%CI)*	p-value*
**Genotype**																		
CC	1.5	1.36	11.48, 2 0.0032	1.86	1.52	9.085, 2 0.0106	1.16	1.11	3.620, 2 0.1637	0.73	0.79	0.3487, 2 0.8400	1.78	1.47	10.53, 2 0.0052	2.8	1.9	15.64, 2 0.0004
	(0.96–2.5)	(0.97–1.9)		(1.01–0.34)	(1.01–2.2)		(0.51–2.6)	(0.6–2.0)		(0.2–2.1)	(0.3–1.8)		(0.64–4.9)	(0.78–2.7)		(1.03–7.55)	(1.11–3.23)	
GC	1.85	1.65		1.77	1.59		2.12	1.82		1.18	1.15		3.76	2.68		3.76	2.68	
	(1.03–3.3)	(1.02–2.6)		(0.85–3.6)	(0.88–2.8)		(0.87–5.15)	(0.9–3.6)		(0.36–3.7)	(0.43–3.05)		(1.3–10.7)	(1.3–5.3)		(1.3–10.7)	(1.3–5.3)	
GG	0.47	0.67		0.4	0.62		0.54	0.74		1.14	1.05		0.19	0.37		0.086	0.18	
	(0.2–0.7)	(0.53–0.85)		(0.2–0.73)	(0.4–0.8)		(0.25–1.15)	(0.5–1.1)		(0.47–2.7)	(0.7–1.4)		(0.06–0.62)	(0.15–0.89)		(0.02–0.38)	(0.05–0.69)	
**Allele**																		
C	1.81	1.42	13.06, 1 0.0003	2.04	1.51	11.38, 1 0.0007	1.38	1.22	1.421, 1 0.2333	0.81	0.86	0.3609, 1 0.5480	1.58	1.32	6.773, 1 0.0093	4.5	2.06	17.72, 1 < 0.0001
	(1.3–2.5)	(1.17–1.7)		(1.3–3.1)	(1.2–1.9)		(0.8–2.3)	(0.8–1.69)		(0.41–1.5)	(0.54–1.3)		(0.78–3.1)	(0.88–1.9)		(2.1–9.5)	(1.58–2.7)	
G	0.55	0.78		0.48	0.74		0.72	0.88		1.22	1.06		0.63	0.83		0.22	0.45	
	(0.3–0.7)	(0.6–0.8)		(0.3–0.7)	(0.6–0.8)		(0.4–1.2)	(0.7–1.09)		(0.6–2.3)	(0.8–1.2)		(0.3–1.2)	(0.6–1.13)		(0.1–0.4)	(0.27–0.75)	

^**a**^OR = Odd ratio,

^**b**^RR = Risk ratio

CI = Confidence interval

χ2 = chi-square; df = Degree of freedom

*p-value is showing statistical significance of chi-square based on frequency distribution given in Table 2 while *p*-value is considered to be significant if it is less than 0.05

### IL-6 (-174-G/C) polymorphism affecting the expression of SOCS3 and IL-6

**Fig 1:**
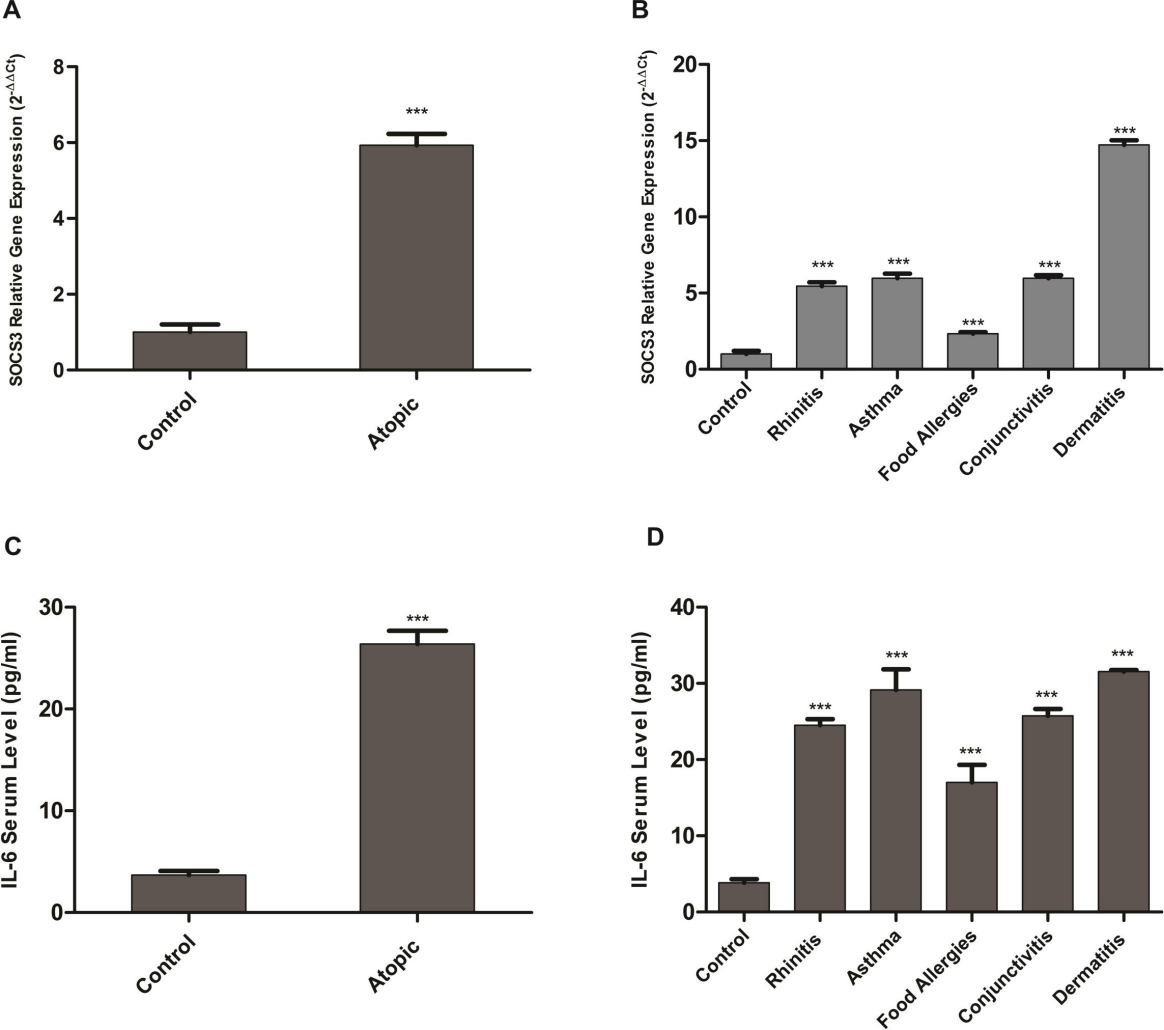
SOCS3 expression and IL-6 serum levels in the control and atopic cases. (A) SOCS3 was up-regulated in allergic/atopic cases in comparison to controls. (B) A general trend of up-regulation of SOCS3 has been observed in all atopic conditions. (C) The atopic cases had significantly higher IL-6 in their serum (*p*-value = 0.0023) in comparison to control population. (D) IL-6 serum levels were elevated in all atopic conditions. The elevated IL-6 serum levels in cases with asthma and dermatitis indicated progressive inflammation. Patients with rhinitis (Mean±SD = 24.5±1.9 pg/ml, *p*-value < 0.0001), asthma (Mean±SD = 29.15±5.4 pg/ml, *p*-value < 0.0001), food allergy (Mean±SD = 17 ± 3.99pg/ml, *p*-value < 0.0001), conjunctivitis (Mean±SD = 25.75 ± 1.27pg/ml, *p*-value < 0.0001) and dermatitis (Mean±SD = 31.5 ± 0.5pg/ml, *p*-value < 0.0001) also had significantly higher IL-6 levels in comparison to controls. Data is expressed in terms of mean ± SD. Un-paired student *t* test was performed to calculate significant difference among atopic and control cases “***” indicate *p*-value less than 0.05.

**Fig 2:**
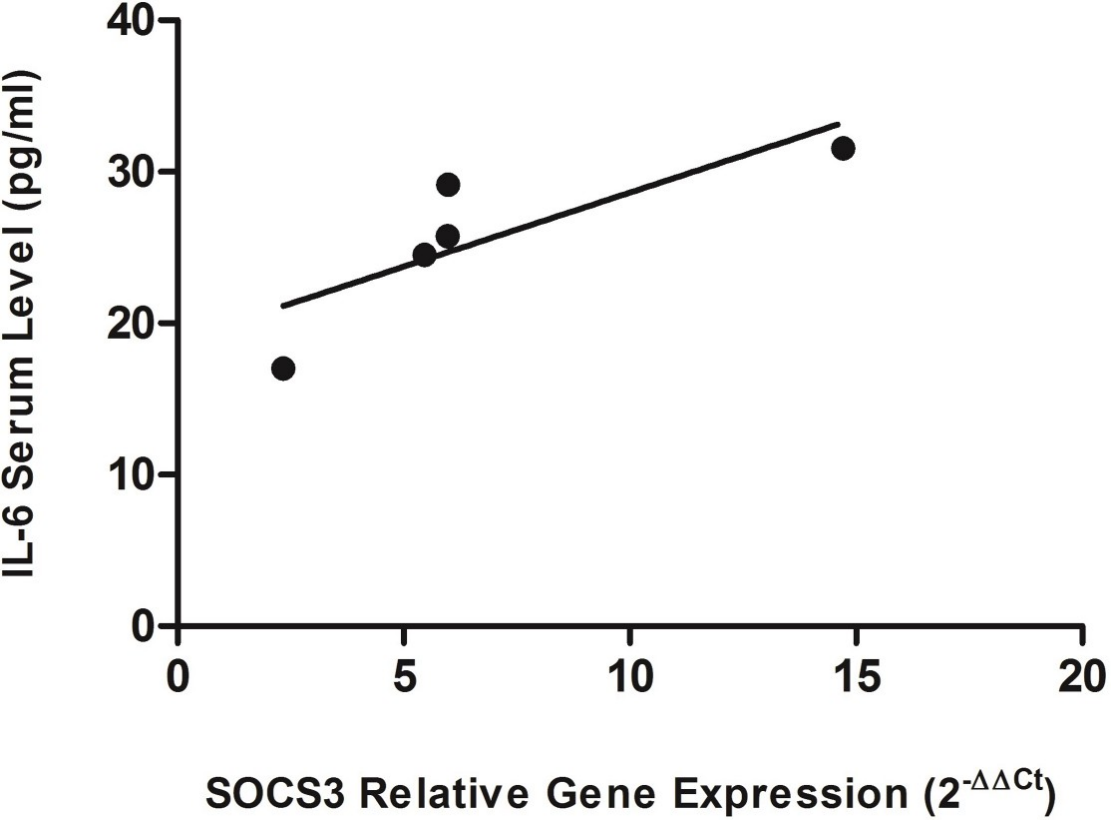
Correlation between IL-6 serum levels and SOCS3 expression. IL-6 and SOCS3 have shown strong correlation (*r* = 0.81). This correlation indicates that SOCS3 expression is tightly regulated by serum IL-6 levels. Data is expressed in terms of mean ± SD. Pearson correlation was performed to calculate significant difference among atopic and control cases. *r* value was taken as measure for correlation.

**Fig 3:**
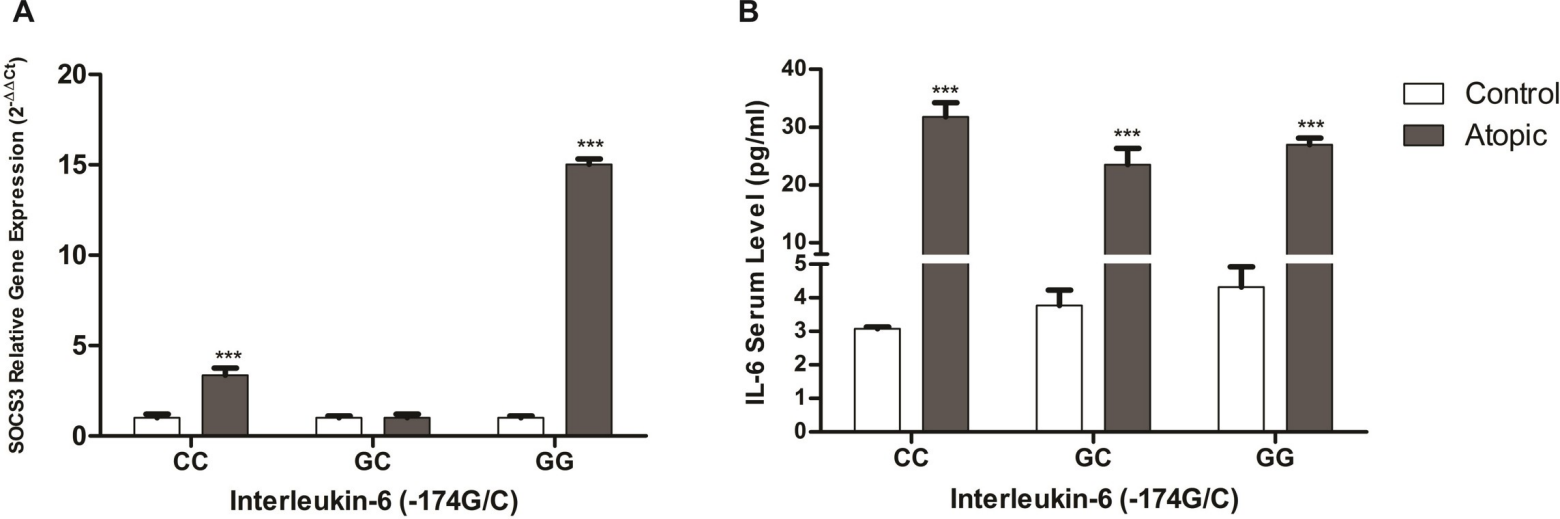
Influence of IL-6 (-174-GC) polymorphism on IL-6 serum levels and the expression of SOCS3. (A) The patients with GG and CC genotype had significant up-regulation of SOCS3 expression whereas SOCS3 expression was equivalent to controls in patients with GC genotype. (B) Despite being the low producing genotype, atopic cases with CC genotype had significantly higher IL-6 serum levels. However, the atopic cases with GG and GC genotype had elevated IL-6 serum levels. The control cases depicted a linear trend in IL-6 expression, control cases with CC genotype had IL-6 serum levels at the lower basal level whereas the control cases with GG genotype had IL-6 levels at higher basal level. The control cases with GC genotype had moderate IL-6 expression. Data is expressed in terms of mean ± SD. Un-paired student *t* test was performed to calculate significant difference among atopic and control cases “***” indicate *p*-value less than 0.05.

IL-6 (-174-G/C) polymorphism has been found to affect expression of both IL-6 and SOCS3. In comparison to control cases SOCS3 levels were significantly higher in the cases with atopy (mean fold change = 5.93 ± 0.2, *p*–value = <0.0001) Fig 1A. Further analysis revealed the significant elevation of SOCS3 levels in each allergic sub-group; asthma (mean fold change = 5.97 ± 0.3, *p*–value = <0.0001), rhinitis (mean fold change = 5.46 ± 0.25, *p*–value = <0.0001), dermatitis (mean fold change = 14.7 ± 0.3, *p*–value = <0.0001), conjunctivitis (mean fold change = 5.97 ± 0.2, *p*–value = <0.0001) and food allergy (mean fold change = 2.3 ± 0.1, *p*–value = <0.0001). The up-regulation of SOCS3 was evident in each type of allergy in comparison to the control cases as shown in Fig 1B. This up-regulation of SOCS3 provides insight to the prognosis of allergy. The IL-6 serum levels corroborated with previous findings and were significantly elevated in atopic cases (Mean±SD = 26.4 ± 5.64 pg/ml, *p*–value = <0.0001) as compared to control cases (Mean±SD = 3.68 ± 1.29 pg/ml) (Fig 1C) [27]. In allergic sub-groups a trend similar to SOCS3 expression was observed Fig 1D. Thus confirming IL-6 elevation that leads to increased SOCS3 expression. We further evaluated the dependence of SOCS3 expression on IL-6 levels. The results indicated a strong correlation between IL-6 serum levels and SOCS3 expression (*r* = 0.81) Fig 2. Furthermore, a significant association between IL-6 (-174-G/C) polymorphism and SOCS3 expression in control and atopic cases was observed. The cases, both allergic and control, with GG and GC genotype had higher IL-6 serum levels which is in accordance with the literature Fig 3B [27]. However, the allergic cases with CC genotype had significantly elevated IL-6 serum levels (Mean±SD = 31.78± 7.36 pg/ml *p*–value = <0.0001) whereas the control cases with CC genotype had lower IL-6 serum levels. The allergic cases with GG genotype depicted increased SOCS3 expression (mean fold change = 15 ± 0.3, *p*–value = <0.0001) in comparison to patients with CC and GC genotypes (Fig 3A). The low risk genotype, GG, depicted significantly increased SOCS3 expression but the high risk heterozygous genotype, GC, had SOCS3 expression (mean fold change = 1.01 ± 0.2, *p*–value = 0.74) similar to the control population and lower than the other two polymorphic genotypes. However, no significant difference in the expression of SOCS3 was observed in the heterozygous, GC, genotype when compared with control cases. The CC genotype had elevated SOCS3 levels (mean fold change = 3.36 ± 0.4, *p*–value = <0.0001) than control population thus confirming the risk for the prognosis of the allergic conditions. Furthermore, IL-6 serum levels were significantly elevated in the atopic cases in comparison to the control cases (*p*–value = 0.0023) and corroborated with the expression of SOCS3 in respective atopic groups (Fig 1A and 1B). As indicated in Fig 3A, the CC genotype had significantly increased SOCS3 expression but IL-6 serum levels were higher in these cases in comparison to other two, GG and GC, genotypes. However, the expression of SOCS3 was equivalent to control cases despite of high IL-6 serum level, thus indicating altered IL-6/SOCS3 axis. Taken together IL-6 and SOCS3 are interdependent and are elevated in allergic conditions and vary in respective genotypes.

### Gender biasness in allergy

**Fig 4:**
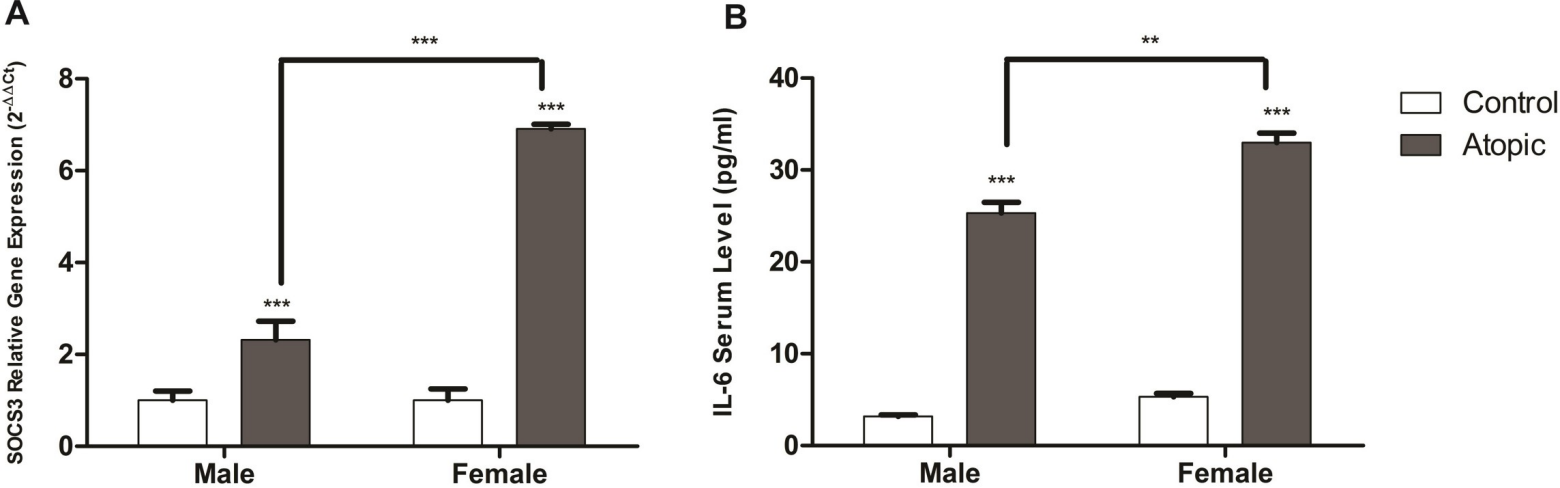
Comparison of SOCS3 and IL-6 serum levels between male and female. (A) The up-regulation of SOCS3 has been observed in the atopic male and female cases. However, the females had elevation in SOCS3 levels that was indicative of higher chances of allergy in females. (B) Elevate IL-6 serum levels have been observed in female atopic cases in comparison to male atopic cases (*p-*value = 0.0066). Data is expressed in terms of mean ± SD. Un-paired student *t* test was performed to calculate significant difference among atopic and control cases. *p*-value less than 0.05 was considered significant.

The allergic cases were relatively prevalent in the female population (Table 1). The expression of SOCS3 in female allergic cases was around 6.91 ± 0.1 mean fold change that is significantly higher than the male allergic cases (*p*–value = <0.0001) (Fig 4A). Moreover, the serum IL-6 levels were significantly elevated in female allergic cases than male allergic cases (*p*–value = 0.0066) (Fig 4B), thus signifying the gender biasness of allergic prognosis. The results signified the role of SOCS3 in allergic reaction as it was elevated in different types of allergies under the influence of IL-6 serum levels.

## Discussion

IL-6 is a potent regulator of allergic reactions via Th2 response. Studies have shown that IL-6 inhibits Th1 response while promotes Th2 cell differentiation via IL-4 induction. Moreover, it may be a determinant factor for Th2 response during asthma and other allergic conditions [41, 42]. The binding of IL-6 to receptor initiates downstream signaling of SOCS3, the auto-inhibitor of IL-6, has been widely studied in allergy [43]. The increased SOCS3 levels in CD3^+^ T cells of allergic patients have been observed [34]. IL-6 (-174-G/C) polymorphism has been associated with altered serum levels of IL-6, which is responsible for inflammatory reaction and activation of STAT3-SOCS3 axis. IL-6/STAT3/SOCS3 axis is interdependent, up-regulation or down-regulation of any molecule alters the cellular response [20]. SOCS3 is elevated in the case of allergic conditions as it is regulated by IL-6 serum levels and lower expression of SOCS3 may lead to the severity of the disorder [26, 44]. Studies have shown elevated SOCS3 levels in the case of allergic rhinitis [45]. Increased SOCS3 and IL-6 expression in atopic conditions such as asthma, rhinitis, dermatitis, conjunctivitis and food allergies play pivotal role in the development of atopic conditions.

The IL-6 (-174-G/C) polymorphism is associated with the alteration of IL-6 serum levels [27]. Our study depicted a pattern of IL-6 serum level alteration in different study groups and their respective genotypes. The low risk high IL-6 producing genotype, GG, depicted increased SOCS3 levels thus implying that it has a protective effect against the allergic conditions. The SOCS3 molecule is known to affect T-cell differentiation thus making it a molecule of extreme importance [46]. The study confirms the protective role of SOCS3 in the atopic conditions as the low risk genotype or genotype with protective effect had elevated SOCS3 levels. This may be due to the higher IL-6 serum levels in the serum of the atopic cases with GG genotype. Despite of being the low producing genotype, CC, had relatively increased SOCS3 levels but the OR value established its role as a risk genotype for allergy. However, in atopic cases with CC genotype had highest IL-6 serum levels among all three genotypes. This may be an indication for an altered signaling crosstalk during allergic conditions. Studies have confirmed IL-6 elevation and its altered signaling network in different atopic cases [47–49]. The atopic cases with heterozygous genotype, GC, had increased IL-6 serum level but SOCS3 expression was at basal level that is equivalent to control population, thus indicating a malfunction in IL-6/SOCS3 axis. Studies have shown that overexposure of IL-6 ameliorates SOCS3 expression and activity [26, 27]. Although, no significant difference was observed in the distribution of homozygous and heterozygous genotypes of IL-6 (-174-G/C) polymorphism but GG acted as a prevalent genotype with a protective effect against allergy. Studies have explicitly described the prevalence of homozygous genotypes among the allergic population [29, 31].

Moreover, studies depicting the variation in SOCS3 expression in different atopic cases indicated the progression of allergy when SOCS3 molecule is unable to produce its effect [26]. The elevated SOCS3 levels in females with atopic condition make it an important prognostic marker for allergy. The cases of allergy are more prevalent in women after puberty this is probably due to the hormonal changes [50]. Notably higher IL-6 serum levels were observed in female atopic cases than in male atopic cases. Female atopic cases presented with higher IL-6 levels in comparison to males. As a consequence difference in the expression of SOCS3 among male and female patients signified the active involvement of SOCS3 in allergic process. Furthermore, the expression of SOCS3 is tightly regulated by the activity of IL-6 [20]. The higher level of both SOCS3 and IL-6 in atopic conditions ensured their crosstalk in the defense signaling. Between high and low producing genotypes, former showed marked increase in SOCS3 mRNA level therefore it can be implied that IL-6 (-174-G/C) polymorphism has many aspects to be explored yet. Furthermore, the previous studies have indicated high producing genotype (GG) being linked to severity of the allergy [29, 31]. Despite being the low producing genotype, atopic cases with CC genotype had elevated IL-6 serum levels. As the study has established CC as the risk genotype so there may be some alteration in IL-6/SOCS3 axis that might lead to allergic severity. In comparison to IL-6 serum levels the SOCS3 expression level increased as it was elevated in atopic cases with GG genotype. Studies have shown elevated IL-6 levels and a protective role of SOCS3 in allergic inflammation [25, 26]. SOCS3 has been reported as a prognostic and diagnostic marker as its expression affects the prognosis of various diseases [26]. SOCS3 has been evaluated for its role in the prognosis of allergy and other inflammatory conditions [19, 26].

IL-6 (-174-G/C) polymorphism has been evaluated for its association with various inflammatory conditions such as rheumatoid arthritis (RA), hepatitis C (HCV) and allergy [27, 29, 32]. The GG genotype has been associated with the higher production of IL-6 along with being established as a risk genotype for various inflammatory conditions [27, 31]. IL-6/SOCS3 axis plays a pivotal role in the prognosis of inflammatory diseases and SOCS3 has been considered to be an active biomarker for allergic conditions [35]. Thus, the increased expression of SOCS3 in allergic conditions in correlation with the IL-6 (-174-G/C) polymorphism signify that it could be a potential biomarker. The variation of SOCS3 expression between allergic men and women indicate the vital role of SOCS3 during the allergic process. The variation in the SOCS3 expression in different types of allergies and the expression difference between male and female establish it as a reliable candidate for biomarker to determine the prognosis of allergy. Moreover, the association between GG and SOCS3 expression implied that SOCS3 expression is firmly regulated by IL-6 serum levels thereby making IL-6 as a potential candidate involved in the prognosis and pathogenesis of allergy. Broadly, the study established the protective role of SOCS3 as it was influenced by the high producing genotype, GG.
